# *De Novo* mutation of FOXF1 causes alveolar capillary dysplasia with misalignment of pulmonary veins: A case report

**DOI:** 10.1097/MD.0000000000025375

**Published:** 2021-04-09

**Authors:** Lili Deng, Xingzhu Liu, Jieqing Min, Zhongjian Su, Yanfei Yang, Liping Ge, Zuozhen Yang, Bin Li, Xing Zhang

**Affiliations:** a Department of Cardiology, Kunming Children's Hospital, Kunming, Yunnan; b Cipher Gene LLC, Beijing, China.

**Keywords:** ACD/MPV, FOXF1, pathogenic mutation, pulmonary hypertension, respiratory distress

## Abstract

**Rationale:**

Alveolar capillary dysplasia with misalignment of the pulmonary veins (ACD/MPV) is a rare congenital malformation in neonates that results in severe respiratory distress and pulmonary hypertension. ACD/MPV is caused by mutations in the FOXF1 gene. Herein, a new case of a girl with ACD/MPV carrying a novel pathogenic variant of FOXF1 was reported.

**Patient concerns:**

A 3-month-old Chinese girl was admitted to the hospital presenting a complaint of cyanosis for 10 days and respiratory distress for 2 days. The history of foreign body inhalation was denied.

**Diagnoses:**

Blood routine, liver and kidney function, electrolytes, type B natriuretic peptide, electrocardiogram, cardiac computed tomography (CT), and echocardiography were done after admission. Dysplasia of the alveolar and the left upper pulmonary vein was displayed through cardiac CT. Echocardiography showed atrial septal defect, tricuspid valve malformation, and pulmonary hypertension. Sequence analysis of FOXF1 from genomic deoxyribonucleic acid (DNA) revealed that the patient was heterozygous for a novel missense variant (c.418 C>T, p.Pro140Gly). Furthermore, genetic analysis of both parents confirmed the de novo occurrence of the variant. Conservation analysis showed that the locus was highly conserved across species. Then, ACD/MPV was a clinical diagnosis.

**Interventions:**

After admission, nasal catheter oxygen inhalation, cefazoxime sodium, furosemide diuretic, milrinone lactate, and Bosentan were given to the patient.

**Outcomes:**

After 6 days of hospitalization, the patient's condition did not improved, the parents gave up treatment and discharged. The patient died half a month after discharge.

**Lessons:**

ACD/MPV is a rare congenital malformation with a poor prognosis. A new *de novo* mutation of FOXF1 was found in our case. Non-invasive methods such as DNA sequencing and FOXF1 analysis are helpful in the clinical diagnosis of ACD/MPV especially in early infants with respiratory distress and pulmonary hypertension.

## 1 Introduction

Alveolar capillary dysplasia with misalignment of pulmonary veins (ACD/MPV) is a rare congenital malformation in newborns arising due to abnormalities in the air-blood barrier of the lung, decrease in the number of functional air-blood barriers, and impaired gas exchange. All these dysfunctions result in severe respiratory distress and pulmonary hypertension.^[1]^

FOXF1—the pathogenic gene for ACD/MPV was identified in a 2009 study on 10 patients with ACD/MPV^[2]^; nonsense, stop-loss, and frameshift mutations were observed. Several studies have also reported copy number variation in the region covering FOXF1, resulting in ACD/MPV.^[3]^

Here, we present a novel pathogenic FOXF1 missense mutation of ACD/MPV. Besides severe pulmonary arterial hypertension and respiratory distress, the proband also presented with a lung infection, electrolyte disturbance, atrial septal defect, tricuspid valve malformation, patent ductus arteriosus, and weight loss.

## 2 Case presentation

**Figure 1:**
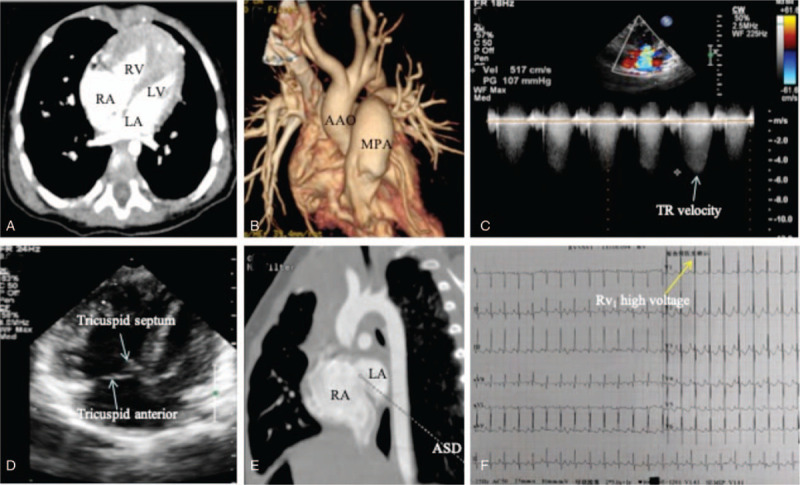
Clinical features of the proband. (A) RA and RV were significantly enlarged; the RV wall exhibited marked hypertrophy. (B) MPA was markedly dilated; the diameter ratio of MPA to AAO was 1.5. (C) Pulmonary artery pressure was estimated to be 117 mmHg with tricuspid regurgitation. (D) A dislocation in closure of the tricuspid valve because of redundant anterior leaflet and dysplasia of septal leaflet. (E) ASD existed between the RA and the LA. (F) RV1 was upright with high voltage indicating an increase in right ventricular pressure load. AAO = ascending aorta, ASD = atrial septal defect, MPA = main pulmonary artery, RA = right atrium, RV = right ventricle, RV1 = R-wave of lead V1, TR = tricuspid regurgitation.

A 3-month-old Chinese girl was admitted to our hospital for pulmonary infections and poor breastfeeding. The parents of the child reported no symptoms or growth retardation, and there was no family history of pulmonary hypertension. A 2/6 systolic murmur was heard at the lower right sternal border. The second heart sound of the pulmonary valve was significantly enhanced. Wet rales were heard in both lungs. In comparison to other pulmonary hypertension features, she presented with more common clinical features such as the following: cardiac insufficiency and elevated type B natriuretic peptide of 440 pg/ml (ref: <100 pg/ml). Dysplasia of the alveolar and the left upper pulmonary vein was displayed through cardiac computed tomography (CT). Echocardiography and cardiac CT both showed signs of pulmonary hypertension: right atrium and right ventricle enlargement, right ventricular wall thickening, and main pulmonary artery dilation (ratio of the main pulmonary artery diameter/inner diameter of the ascending aorta = 1.39, ref: <1.2). High voltage in the right ventricle was observed on an electrocardiogram (ECG) (Fig. 1). She also exhibited characteristic clinical conditions such as electrolyte disturbance, lung infection, liver dysfunction, and tricuspid valve malformation (Fig. 1) (Table 1)

**Table 1:** Coexisting clinical conditions with ACD/MPV.

S. no.	Clinical condition	Particulars
1	Lung infection	WBC 14.01×10^9/L,L%63.20%, PLT 598.00×10^9/L CRP 2.02mg/L (ref: 0–10 mg/L)
2	Moderate liver dysfunction	ALT 41U/L (ref: 5–40 U/L); AST 59U/L (ref: 8–40 U/L)
3	Electrolyte disturbance	Low blood sodium: 132mmo/l (ref:135–145 mmol)
High blood potassium: 6.1 mmo/l (ref:3.5–5.5 mmol)		
4	Atrial septal defect	Diameter: 8.0 mm
5	Tricuspid valve malformation	Closure dislocation exists because of the redundant anterior leaflet and dysplasia septal leaflet of the tricuspid valve, which results in severe tricuspid regurgitation (the reflux area is about 3.3 cm^2^)
6	Electrocardiogram	RV1 is upright with high voltage (ref: Inverted)

ACD/MPV = alveolar capillary dysplasia with misalignment of the pulmonary veins.

## 3 Diagnostic assessment

Peripheral blood samples were collected from the proband and family members. Genomic deoxyribonucleic acid (DNA) was extracted and a total of 1.0 μg genomic DNA per sample was used as input material for DNA sequencing. A total of 895 genes associated with cardiovascular and other related diseases were selected by a gene capture strategy, using the GenCap custom enrichment kit (MyGenostics Inc., Beijing, China) following the manufacturer's protocol. Sequencing was performed on the Illumina Novaseq 6000 platform.

Burrows–Wheeler Aligner was utilized to map the paired-end clean reads to the human reference genome (hg19).^[5]^ GATK^[6]^ was utilized to recalibrate the base quality score, single nucleotide polymorphisms (SNP), and short indel calling. The variations were annotated using ANNOVAR.^[7]^ Variants were picked up in the exonic and splicing regions with a minor allele frequency of ≤0.005 in the SNP database (ExAC_EAS, ExAC_ALL, 1000 Genomes, gnomAD). The identified variants were confirmed by segregation analysis and further validated using Sanger sequencing.

**Figure 2:**
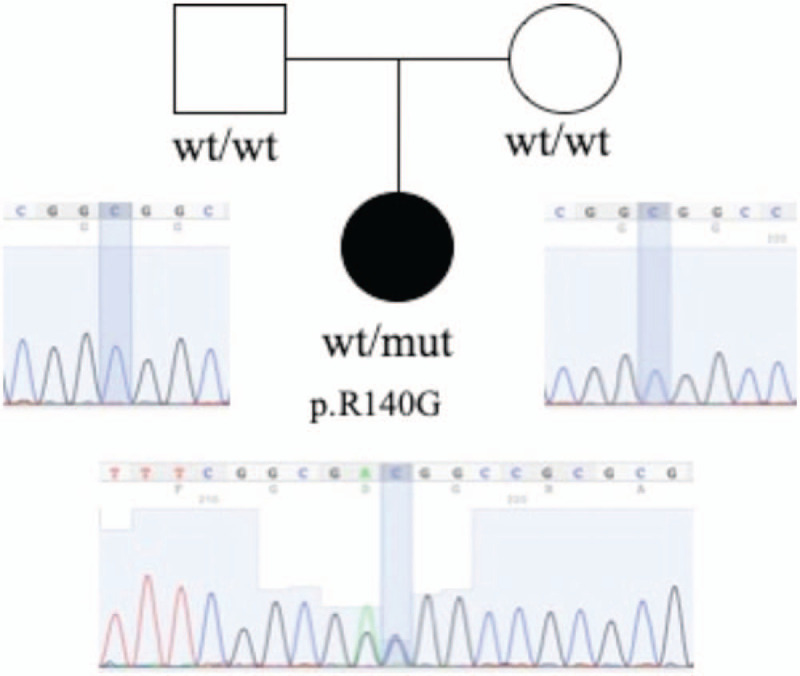
The genogram of the patient's family; the variant of FOXF1 (NM_001451, c.418 C>T, and p.Pro140Gly) in proband and parents. mut = mutation, wt = wild type.

**Figure 3:**
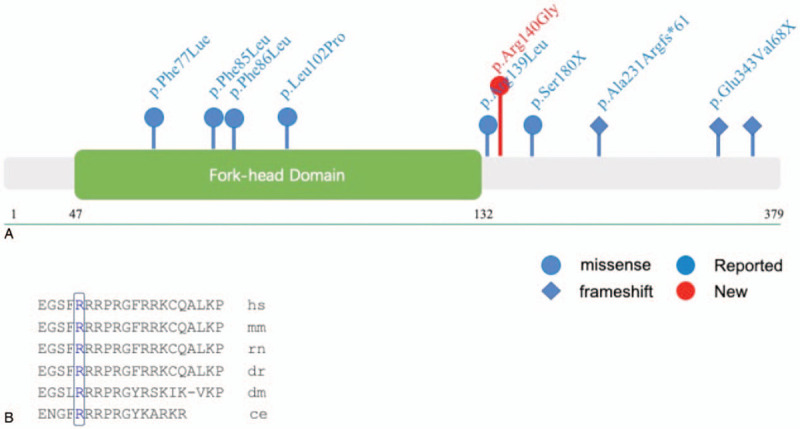
Missense mutation and splicing variations in FOXF1. (a) Functional domains and mutations in the FOXF1 gene as indicated. Blue: reported mutations; red: *novo* mutation found in the patient. Dots: missense mutation; Diamond: frameshift mutation. (b) Evolutionary conservation of the proline residues at position 418 in the FOXF1 gene across different species.

The child carried a novel mutation (418 C>T transitions, resulting in 140 Pro-to-Gly substitutions) in the FOXF1 gene; the parents, however, had the FOXF1 wild-type gene. Sanger sequencing confirmed this variant (Fig. 2). The variant was located in the first exon near the end of the fork head domain of FOXF1; variant data was not available in the curated databases—1000 Genomes, ExAC, and gnomAD. It was predicted to be deleterious using the bioinformatic tools—Polyphen2, Mutation, taster, and SIFT. As per the American College of Medical Genetics and Genomics and the Association for Molecular Pathology variant interpretation guidelines, the variant was likely to be pathogenic.^[4]^ Furthermore, conservation analysis showed that the locus was highly conserved (Fig. 3).

Informed consent was obtained from the parents and their families. The patient underwent a careful examination. This study was approved by the hospital.

## 4 Discussion

Here, we report the case of a Chinese girl with ACD/MPV carrying a novel *de novo* variant of FOXF1. She showed respiratory distress accompanied by severe pulmonary hypertension, lung infection, electrolyte disturbance, atrial septal defect, tricuspid valve malformation, and weight loss. Based on her clinical features, we suspected a complex respiratory and cardiovascular system syndrome, perhaps ACD/MPV; hence, we performed sequencing of the targeted region and analyzed for the presence of potentially pathogenic mutations. We eventually observed a missense variation in the FOXF1 gene.

This is the first-ever report of a *de novo* (c.418 C>T, p.Pro140Gly) variant observed in the FOXF1 gene of the patient in this study. However, a pathogenic c.416G>T variant has been reported previously, which results in a p.Arg139Leu change.^[8]^ The mutation data was not available in the 1000 Genomes, ExAC, and gnomAD databases. It was predicted to be deleterious as well as a novel variation in the family by Polyphen2, Mutation taster, and SIFT tools. We therefore concluded that this variant is likely to be pathogenic and the cause of ACD/MPV in the patient. It was very helpful in the rapid diagnosis of multiple organ dysfunction syndrome and its noninvasive prognosis.

FOXF1 belongs to the FOX transcription factor family and is characterized by a conserved DNA-binding domain.^[9]^ The missense variant identified in our study was near the end of the fork-head domain of FOXF1; previous studies have demonstrated a crucial role of the domain in the role of transcription factors.^[10]^ Evolutionary conservation analysis shows the mutation locus to be highly conserved.

There are several challenges in the diagnosis of ACD/MPV. Clinical features of the reported cases were heterozygous^[11]^ and could coexist with colobomas, hemi hyperplasia, duodenal stenosis, or intestinal malrotation. It can often be misdiagnosed or undiagnosed by clinicians, especially due to the lack of clinical knowledge about ACD/MPV. Previously, diagnosis depended on the pathologic features of lung tissue autopsy or antemortem lung biopsy; however, these methods have not been applied clinically. Fortunately, FOXF1 gene testing can provide an accurate and non-invasive alternative for clinicians.

To conclude, we identified a novel, likely pathogenic variant of FOXF1, possibly causing ACD/MPV. The clinical features of the proband included additional anomalies and complex syndromic phenotypes compared to other reports. Non-invasive methods such as DNA sequencing and FOXF1 analysis are helpful in the clinical diagnosis of ACD/MPV.

## Acknowledgments

We thank the proband and her family members for their kind co-operation as well as Ciphergene LCC for data mining and support in finalizing the manuscript. We would like to thank Editage (www.editage.com) for English language editing.

## Author contributions

LLD, XZL, JQM, and XZ carried out the studies, participated in collecting the data, and drafted the manuscript. YFY, LPG, and ZZY performed the statistical analysis and participated in its design. ZJS and XZ helped to draft the manuscript. All authors read and approved the final manuscript.

**Conceptualization:** Zhongjian Su, Yanfei Yang, Bin Li, XING ZHANG.

**Data curation:** Zhongjian Su, Liping Ge, Zuozhen Yang, Jieqing Min.

**Formal analysis:** Liping Ge, Jieqing Min.

**Investigation:** Zhongjian Su, Jieqing Min.

**Methodology:** Yanfei Yang, Jieqing Min.

**Software:** Zuozhen Yang.

**Supervision:** Bin Li, XING ZHANG, Jieqing Min.

**Validation:** XING ZHANG.

**Writing – original draft:** Lili Deng, Xingzhu Liu, XING ZHANG.

**Writing – review & editing:** Lili Deng, Xingzhu Liu, Bin Li, XING ZHANG.
